# Preparation of Covalent-Ionically Cross-Linked UiO-66-NH_2_/Sulfonated Aromatic Composite Proton Exchange Membranes With Excellent Performance

**DOI:** 10.3389/fchem.2020.00056

**Published:** 2020-02-18

**Authors:** Penglun Zheng, Quanyi Liu, Donghui Wang, Zekun Li, Yawei Meng, Yun Zheng

**Affiliations:** ^1^College of Civil Aviation Safety Engineering, Civil Aviation Flight University of China, Guanghan, China; ^2^Key Laboratory of Optoelectronic Chemical Materials and Devices, Ministry of Education, Jianghan University, Wuhan, China

**Keywords:** metal-organic frameworks, sulfonated poly(arylene ether nitrile)s, proton conductivity, selectivity, methanol permeability

## Abstract

Metal-organic frameworks (MOFs), as newly emerging filler materials for polyelectrolytes, show many compelling intrinsic features, such as variable structural designability and modifiability of proton conductivity. In this manuscript, UiO-66-NH_2_, a stable MOF with -NH_2_ functional groups in its ligands, was selected to achieve a high-performance sulfonated poly(arylene ether nitrile)s (SPENs)/UiO-66-NH_2_-x covalent-ionically cross-linked composite membrane. Simultaneously, the obtained composite membranes displayed excellent thermal stability and dimensional stability. The as-prepared SPEN/UiO-66-NH_2_-x cross-linked membranes exhibited higher proton conductivity than recast SPENs, which can be attributed to the construction of ionic clusters and well-connected ionic nanochannels along the interface between UiO-66-NH_2_-x and SPEN matrix via molecular interactions. Meanwhile, the methanol permeability of the SPEN/UiO-66-NH_2_-x composite membrane had been effectively reduced due to the barrier effect of cross-linking and the addition of UiO-66-NH_2_-x. The SPEN/UiO-66-NH_2_-5 composite membrane had the highest selectivity of 6.42 × 10^5^ S·s·cm^−3^: 14.3-times higher than that of Nafion 117. The preparation of cross-linked UiO-66-NH_2_/SPEN composite was facile, which provides a new strategy for preparing high performance proton exchange membrane.

## Introduction

Direct methanol fuel cell (DMFC) is one of the most eco-friendly power sources because of its relatively high efficiency, clean and low carbon technology, and renewable methanol compared with traditional fossil fuels (Antolini, 2018; Eris et al., 2018; Li et al., 2018a,b; Yilmaz and Can, 2018). Proton exchange membranes (PEMs) have attracted substantial attention as the core components of DMFC for ion conduction. Perfluorosulfonic acid (PFSA) polymer membranes are the most widely commercialized proton exchange membrane, due to their high proton conductivity (10^−2^−10^−1^ S·cm^−1^) and excellent chemical stability (Chang et al., 2013; Kuo et al., 2018; Yan et al., 2018; Ling et al., 2019). Among all kinds of proton exchange membranes, Nafion membranes, typically made by DuPont™, has been extensively used owing to their high proton conductivity and good chemical stability. However, Nafion has its disadvantages, such as high cost, low thermal stability, low proton conductivity under high temperature (>80°C), and hazardous manufacturing processes, which restrict its commercial application (Wang et al., 2015a; Zakil et al., 2016; Ressam et al., 2018). In this regard, numerous efforts have been paid to solve these problems. Alternatives or modifications and inorganic–organic composite membranes have been prepared to replace Nafion for exploring new types of PEMs. With this development trend, large quantities of sulfonated aromatic polymers have been explored as alternative PEMs to Nafion, such as sulfonated poly(arylene ether nitrile)s (Zheng et al., 2017a,b, 2018; Feng et al., 2018), sulfonated polyimides (Perrot et al., 2011; Yin et al., 2011; Yao et al., 2015; Zhang et al., 2017), sulfonated poly(benzimidazole)s (Li et al., 2009; Yue et al., 2015, 2016; Singha et al., 2016), sulfonated poly(arylene ether ketone)s (Pang et al., 2015; Xu et al., 2015; Nguyen et al., 2016; Oh et al., 2016), and sulfonated poly(arylene ether sulfone)s (Ko et al., 2015; Kim et al., 2017a,b; Ahn et al., 2018).

Among all the mentioned sulfonated polymers, SPENs possess great potential as excellent PEMs because of the following striking features (Zheng et al., 2015; Wan et al., 2017; Zhan et al., 2018, 2019): (1) The strong polar nitrile group can promote the adhesion of SPEN to many substrates through strong polar nitrile group interactions with other polar chemical groups; (2) the strong dipole interaction between the copolymer chains leads to good water retention and dimensional stability of SPEN; (3) the excellent chemical and thermal stability and high mechanical strength; and (4) lower cost and the variable structural designability of SPENs. However, for such sulfonated polyaromatic ethers, their dimensional stability, methanol crossover resistance, tensile strength, and thermal stability tend to decrease as the sulfonation degree reaches a high level. The shortcoming of the SPENs might restrict its commercial applications. Cross-linking is recognized as the most efficient method to overcome the aforementioned shortcomings of the SPENs (Zheng et al., 2017c). Unfortunately, cross-linked membranes normally exhibit low proton conductivity due to two factors: (1) Sulfonic acid groups participating in the cross-linked reactions results in the elimination of sulfonic acid groups; (2) the sulfonic acid groups may be diluted by introducing cross-linker. Therefore, besides the cross-linking of the membranes, it is necessary to add proper functional particles to improve the conductivity that can enhance the comprehensive properties of proton exchange membranes.

Metal organic frameworks (MOFs), as newly emerging materials, have attracted great attention in many fields due to their compelling intrinsic structural features, such as large porosity, high crystallinity, tremendous structural flexibility, and pore size tenability as well as tailorable functionality (Wu et al., 2015; Zhang et al., 2015; Zhao et al., 2016; Kahn et al., 2017). In recent years, more and more attention has been paid to the study of proton conduction in MOFs (Gil-Hernández et al., 2015; Liu et al., 2016; Xu et al., 2016; Joarder et al., 2017; Yang et al., 2017). The high crystallinity of MOFs can bring specified proton conduction pathway, such that protons can be transferred through hydrogen bond networks or functional sites loaded in holes. Among them, Zr-MOFs have attracted considerable interests, owing to their high robustness and easy tunable structure. Despite that diverse Zr-MOFs with different structures and porosities have been reported recently (Jiang et al., 2013; Furukawa et al., 2014; Wang et al., 2015b), only the benchmark compound, Zr-terephtalate MOF UiO-66, displays the best performances and the lowest cost of synthesis and scale-up (Cavka et al., 2008; Taddei et al., 2015; DeStefano et al., 2017). Specifically, the metal-ligand bond strength of UiO-66-NH_2_ is stronger than those of MIL-53-NH_2_ and MIL-101-NH_2_ (two other kinds of MOFs with high stability). Thus, to some extent, UiO-66-NH_2_ is relatively more stable compared to MIL-53-NH_2_ and MIL-101-NH_2_ (Bai et al., 2016). However, their proton conduction channels are not consecutive enough and the fabrication of MOFs into the form of membranes is still immature, which restricts their proton conductivity and limits their practical application. The fabrication of MOFs into sulfonated polymer matrix is a significant step for their practical application (Patel et al., 2016; Dong et al., 2017). Sulfonated polymers are not only easy-to-prepare membranes, but also act as a good medium to form continuous and effective channels for proton conduction.

In this manuscript, an amino-functionalized UiO-66 (UiO-66-NH_2_), a well-known Zr-based MOF, was synthesized and *in situ* incorporated with SPENs to form UiO-66-NH_2_/SPENs covalent-ionically cross-linked composite membranes in combination with the high proton conductivity and surface amino reaction activity of UiO-66-NH_2_. The preparation of the cross-linked UiO-66-NH_2_/SPENs composite was facile, which provides a new strategy for preparing high-performance proton exchange membrane. The results showed that the methanol permeability, water uptake, and swelling ratio of the cross-linked UiO-66-NH_2_/SPENs composite membranes decreased with the introduction of functional nanoparticles, which indicates that the covalent-ionically cross-linking between -NH_2_ and carboxyl/sulfonic acid can effectively improve the methanol resistance and dimensional stability of the composite membranes. At the same time, because the -NH_2_ in UiO-66-NH_2_ is a very good proton acceptor/donor, it provides a new way for proton transfer in SPENs membranes, thus significantly improving the proton conductivity of composite membranes. All the results showed that the cross-linked UiO-66-NH_2_/SPENs composite membranes prepared by covalent-ionically cross-linking have excellent comprehensive properties.

## Experiment

### Materials

Hydroquinone sulfonic acid potassium salt (SHQ), hydrochloric acid, zinc powder, 2, 6-difluorobenzonitrile (DFBN) and phenolphthalin (PPL), sodium hydroxide (NaOH, AR), N,N-dimethylacetamide (DMAc, AR), sodium chloride (NaCl, AR), zirconium tetrachloride (ZrCl_4_, AR) 2-aminotropic acid (BDC-NH_2_, AR), acetone (AR), NMP (AR), and K_2_CO_3_ (AR) were supplied by KeLong. All the materials were used without further purification.

### SPEN Synthesis

**Scheme 1:**
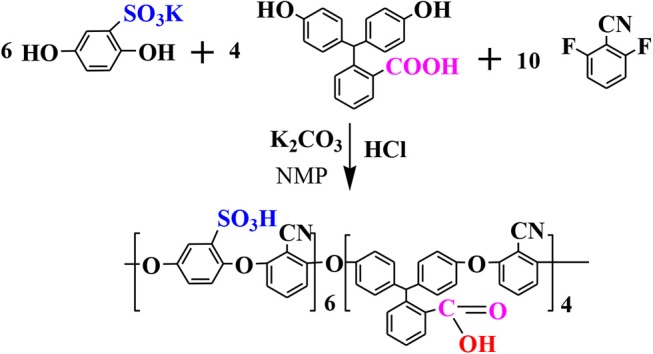
The synthesis of SPEN copolymers via nucleophilic aromatic substitution.

In a typical reaction (Scheme 1), 0.06 mol of SHQ, 0.10 mol of DFBN, 0.2 mol of K_2_CO_3_, 0.04 mol of PPL, 25 mL toluene, and 75 mL NMP were poured into a three-necked flask equipped with a condenser and a mechanical stirrer. The reactants were first heated to 150°C and maintained for 2 h at this temperature. Secondly, it was gradually heated up to 185°C and maintained at this temperature until the copolymers achieve a high viscosity. Finally, the copolymers were precipitated by pouring the mixture into alcohol. The copolymers were thoroughly washed several times for purification, and the collected copolymers were treated at 120°C in vacuum overnight.

### Preparation of UiO-66-NH_2_

Rapid and large-scale synthesis of UiO-66-NH_2_: First, 1L DMF was added into the three-necked bottle equipped with an oil bath, overhead mechanical stirrer, and condenser. The oil bath was raised to 120°C and maintained at this temperature. Then, 15.0 g (64.4 mmol) ZrCl_4_, 11.7 g (64.4 mmol) 2-amino terephthalic acid (BDC-NH_2_), and 440 mL (7.73 mol) acetic acid were added sequentially and stirred to dissolve evenly. The mixture was stirred and maintained at 120°C for 15 min, then naturally cooled to room temperature. The mixture was immersed in DMF for 12 h to remove the unreacted substances, and then immersed in ethanol for 48 h and replaced with ethanol every 6 h. The final product was obtained by drying in a vacuum oven at 120°C.

### Preparation of SPEN/UiO-66-NH_2_-x Covalent-Ionically Cross-Linked Composite Membrane

The SPEN/UiO-66-NH_2_-x covalent-ionically cross-linked composite membranes were obtained as follows: Firstly, a certain amount of SPENs was dissolved in 15 mL DMAc to obtain a clear and transparent solution, which was recorded as solution A. At the same time, a certain amount of UiO-66-NH_2_ was added to the 5 mL DMAc and put into the ultrasonic bath to make it dispersed uniformly, which was recorded as solution B. Then, solution B was poured into solution A, which were sonicated and stirred for several hours at room temperature. The mixed solution then was cast onto clean glass plates and then cured in an oven with the consecutive temperature profiles of 80, 100, 120, 140, and 160°C (2 h each step) to remove the solvent. At the end of the heating procedure, the oven was naturally cooled to room temperature. Then the composite membrane was taken out and immersed in 1 M sulfuric acid solution for 24 h. After that, the membrane was washed repeatedly with DI water to neutralize the residual acid. The composite membranes were then placed in a vacuum oven and treated at 160°C for 12 h to obtain SPEN/UiO-66-NH_2_ covalent-ionically cross-linked composite membranes, named as SPEN/UiO-66-NH_2_-x (x = 0, 1, 3, 5, 7, and 9).

### Characterization

Fourier Transform Infrared (FTIR) spectra were performed by a Shimadzu FTIR8400S spectrometer between 4,000 and 400 cm^−1^ in air. The cross-sectional morphologies of the membranes were observed by scanning electron microscope (SEM, JEOL, JSM-5900 LV). The microstructure, particle size, and element distributions of UiO-66-NH_2_ were characterized by JEM2100F field emission transmission electron microscope (TEM). Before TEM observation, the membranes were brittle fractured in liquid nitrogen and then coated with gold. The thermal degradation processes of the membranes were evaluated by thermal gravimetric analysis (TGA, TA, Q50) with a heating rate of 20°C/min under 40 mL/min N_2_ flow. The sample loading for TGA was around 6–10 mg. The membranes were first heated to 160°C and dwelled for 5 min to remove the remaining water and solvent. They were then reheated from room temperature to 600°C with a heating rate of 20°C min^−1^. The tensile strength and Young's modulus were measured on a SANS CMT6104 series desktop electromechanical universal testing machine at room temperature. The crosshead speed was fixed at 5 mm min^−1^. At least five membrane samples were prepared for the mechanical test.

### Ion Exchange Capacity (IEC)

Weight-based IEC (IEC_w_) was determined by the titration method. All the membranes were immersed in 2.0 M NaCl solution for 48 h to liberate the H^+^ ions. Then, the H^+^ ions were titrated with 0.01 M NaOH solution using phenolphthalein as indicator. The titrated IEC was calculated from the following formula:

$$\begin{array}{l} \text{IEC }\left(\text{mmol}/\text{g}\right)=\frac{{\text{V}}_{\text{NaOH}}^{*}{\text{M}}_{\text{NaOH}}}{{\text{W}}_{\text{dry}}} \end{array}$$

where M_NaOH_ (mol/L) and V_NaOH_ (L) are the concentration and volume of NaOH solution, respectively, and W_dry_ (g) is the mass of membrane.

### Methanol Permeability and Proton Conductivity

The methanol permeability of samples was tested by using a diffusion cell. The cell consists of two diffusion cells (20 mL each) and was separated by the as-prepared membranes. The membranes were hydrated in deionized water for 24 h. Initially, 10 M methanol solution (20 mL) was added in one side of the diffusion cell (cell A), and ultrapure water (20 mL) was added in the other side (cell B). The methanol concentration in the cell B was tested by using a HIMADZU GC-8A chromatograph. The methanol diffusion coefficient was calculated by the following formula:

$$\begin{array}{l} {\text{C}}_{\text{B}}\left(\text{t}\right)=\frac{\text{A}}{{\text{V}}_{\text{B}}}\frac{\text{DK}}{\text{L}}{\text{C}}_{\text{A}}\left({\text{t }-\text{ t}}_{0}\right) \end{array}$$

where L, A, and V_B_ are the thickness of membrane, the effective area, and the volume of receptor reservoir, respectively. C_A_ and C_B_ are the methanol concentrations in the donor and receptor reservoirs, respectively. DK (in cm^2^ s^−1^) denotes the methanol permeability.

The proton conductivity of the membranes was tested from 10^−1^ to 10^6^ Hz. The membrane samples were hydrated in deionized water at different temperatures for 24 h. The membranes were infibulated between two pairs of stainless steel electrodes. Conductivity measurements under fully hydrated conditions were carried out with the stainless steel electrodes immersed in deionized water. The proton conductivity (σ) was calculated by the following formula:

$$\begin{array}{l} \text{σ}=\frac{\text{L}}{\text{SR}} \end{array}$$

where σ is the proton conductivity (S/cm), L is the distance between the electrodes (cm), R is the impedance of the membrane (Ω), and S is the surface area (cm^2^). Each sample was measured three times to ensure data reproducibility.

### Water Uptake and Swelling Ratio

Water uptake was determined by the weight differences between the full-dried and full-hydrated membranes. All membrane samples were immersed in deionized water at different temperatures for 24 h to ensure that the membranes were saturated with water. Subsequently, the water was wiped from the surface of the membranes quickly with blotting paper, and then the weight of wet membranes was measured. The swelling ratio of the membrane sample was determined by immersing it in deionized water at certain temperatures for 24 h and measuring the change in length before and after the swelling process. The water uptake and swelling ratio of the membranes were calculated using the following equations:

$$\begin{array}{l} \text{Water uptake}=\frac{{\text{W}}_{\text{wet}}-{\text{W}}_{\text{dry}}}{{\text{W}}_{\text{dry}}}\times 100\% \end{array}$$

$$\begin{array}{l} \text{Swelling ratio}=\frac{{\text{L}}_{\text{wet}}-{\text{L}}_{\text{dry}}}{{\text{L}}_{\text{dry}}}\times 100\% \end{array}$$

where W_wet_ and W_dry_ are the mass of wet and dry membrane samples, respectively, and L_wet_ and L_dry_ are the length of wet and dry samples, respectively.

## Discussion and Result

### Characterization of UiO-66-NH_2_

**Figure 1:**
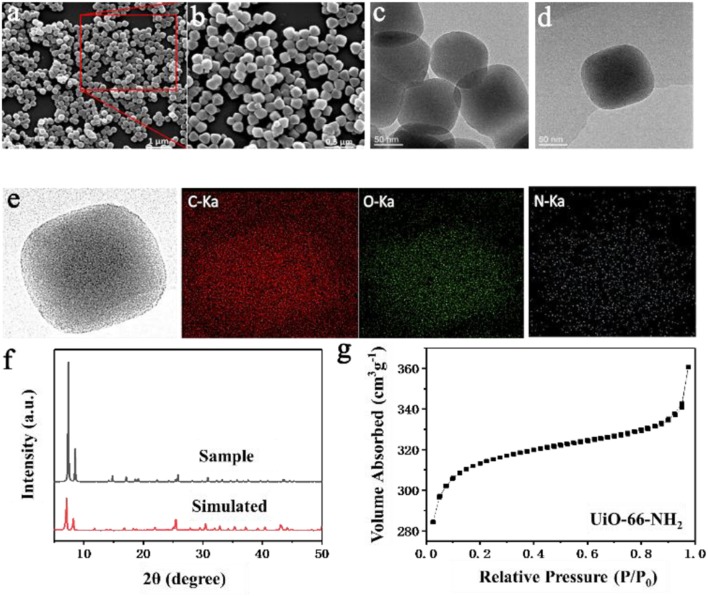
**(a,b)** SEM, **(c,d)** TEM, **(e)** EDS mapping, **(f)** XRD curve, and **(g)** N_2_ adsorption isotherm (77 K) of UiO-66-NH_2_.

The structure, morphology, composition, and porosity of synthesized UiO-66-NH_2_ were studied by various characterization techniques. Firstly, the morphology of synthesized UiO-66-NH_2_ was studied by SEM and TEM, as shown in Figure 1. The synthesized UiO-66-NH_2_ has a highly uniform cubic state with an average diameter of about 135 nm, which can be inferred from Figures 1a,b. The TEM diagrams in Figures 1c,d further confirm the typical cube structure of UiO-66-NH_2_ with smooth and flat surfaces. Figure 1e is the EDS element mapping of UiO-66-NH_2_, which can be used to evaluate the element distribution of synthesized UiO-66-NH_2_ materials. As shown in Figure 1e, the elements of N and O are uniformly distributed in the cubic region corresponding to UiO-66-NH_2_. Figure 1f is the XRD pattern of UiO-66-NH_2_, which is very similar to the simulated curve, indicating the successful synthesis of UiO-66-NH_2_. At the same time, the narrow and sharp diffraction peaks show the high crystallinity of the synthesized UiO-66-NH_2_. The N_2_ adsorption-desorption isotherms of UiO-66-NH_2_ at 77 K are shown in Figure 1g. It is noteworthy that the specific surface area of UiO-66-NH_2_ reaches 1,253.97 m^2^·g^−1^. The large specific surface area can provide enough proton-hopping sites to UiO-66-NH_2_ and enhance the proton transfer in the membrane, which is also conducive to the adsorption of methanol molecules.

### FTIR of SPEN/UiO-66-NH_2_-x Covalent-Ionically Cross-Linked Composite Membrane

**Figure 2:**
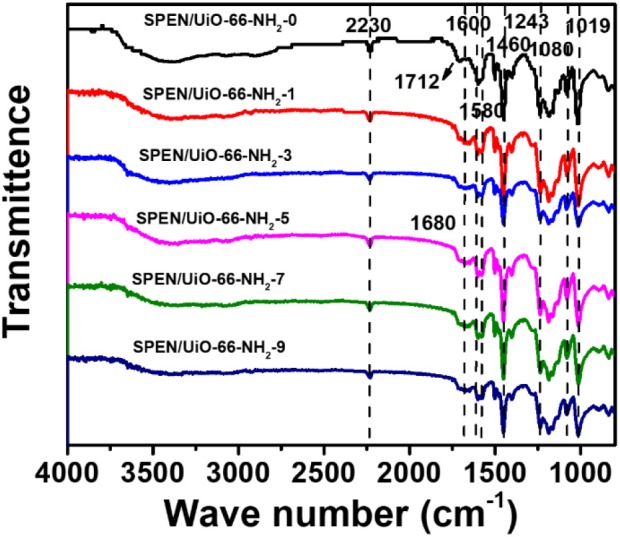
The FT-IR spectra of SPEN/UiO-66-NH_2_-x covalent-ionically cross-linked composite membrane.

The characteristic functional groups in SPEN/UiO-66-NH_2_-x covalent-ionically cross-linked composite membrane were characterized by FTIR. From Figure 2, it can be observed that there is a significant ether bond characteristic absorption at 1,243 cm^−1^ in the composite membranes, which indicates that the sulfonated poly(arylene ether nitrile)s were formed by condensation polymerization. In addition, there is a characteristic absorption band at 2,230 cm^−1^ in all the SPEN/UiO-66-NH_2_-x composite membranes, which is unique to the nitrile group on the main chain of SPEN. The characteristic absorption bands at 1,018 and 1,080 cm^−1^ correspond to the symmetrical and asymmetrical stretching vibration absorption of the sulfonic group (−SO_3_H), which indicates that the −SO_3_H has been successfully introduced into the SPEN. The obvious absorption bands at 1,460, 1,580, and 1,600 cm^−1^ can be attributed to the vibration absorption of benzene ring skeleton. It is also observed that before the addition of UiO-66-NH_2_, there was a distinct characteristic absorption band at 1,712 cm^−1^ for pure SPEN. However, the characteristic absorption band disappeared gradually with the doping of UiO-66-NH_2_, and appeared gradually at 1,680 cm^−1^ which belonged to the characteristic absorption band of carbonyl group, indicating that the -NH_2_ of UiO-66-NH_2_ reacted with the carboxyl groups of SPEN in the SPEN/UiO-66-NH_2_-x composite membranes.

### SEM of SPEN/UiO-66-NH_2_-x Covalent-Ionically Cross-Linked Composite Membrane

**Figure 3:**
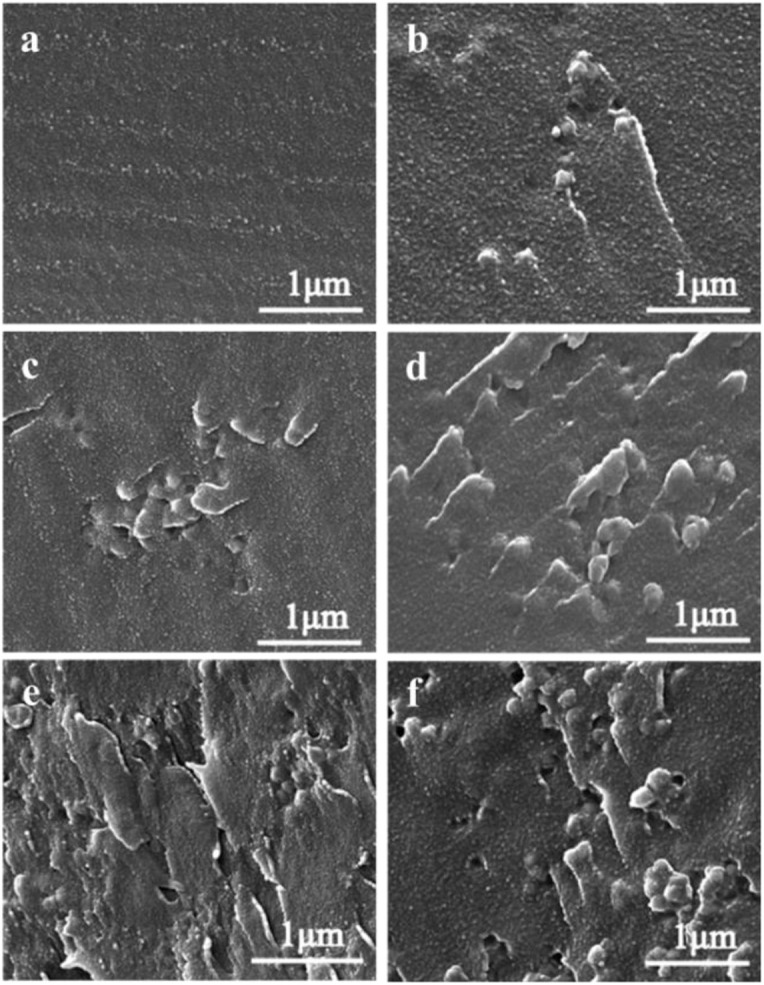
The SEM images of SPEN/UiO-66-NH_2_-x covalent-ionically cross-linked composite membrane with **(a)** x = 0 wt%; **(b)** x = 1 wt%; **(c)** x = 3 wt%; **(d)** x = 5 wt%; **(e)** x = 7 wt%; and **(f)** x = 9 wt%.

The performance of composite membranes is closely related to the dispersion, interface connectivity, and compatibility of functional nanoparticles in the matrix. SEM was used to observe the internal microstructures of the SPEN/UiO-66-NH_2_-x composite membranes and the dispersion of UiO-66-NH_2_ in the membranes. Figure 3 is the SEM image of the cross section of SPEN/UiO-66-NH_2_-x covalent-ionically cross-linked composite membrane. As can be seen in the Figure 3, the SPEN/UiO-66-NH_2_-0 membrane showed a smooth cross section. With the increasing UiO-66-NH_2_ content, the cross section of SPEN/UiO-66-NH_2_-x covalent-ionically cross-linked composite membrane becomes rough. Particularly, some holes appeared in the cross section of SPEN/UiO-66-NH_2_-9 composite membrane. When the doping amount of UiO-66-NH_2_ is no more than 5 wt% (Figures 3b–d), it can be uniformly dispersed in the composite membrane. Moreover, the interface between UiO-66-NH_2_ and SPEN is relatively close, and no phase separation is observed. This can be attributed to the good compatibility of UiO-66-NH_2_ and polymers, and the amino group easily interacts with sulfonic group/nitrile group in SPEN matrix, which greatly increases the compatibility of inorganic function in matrix. However, the agglomeration trend began to appear when the content of UiO-66-NH_2_ further increased, which is well-illustrated in Figures 3e,f. As shown in Figure 3f, when the UiO-66-NH_2_ content reaches 9 wt%, obvious agglomeration was observed. And, some holes begin to appear in the composite membrane.

### Thermal Properties

**Figure 4:**
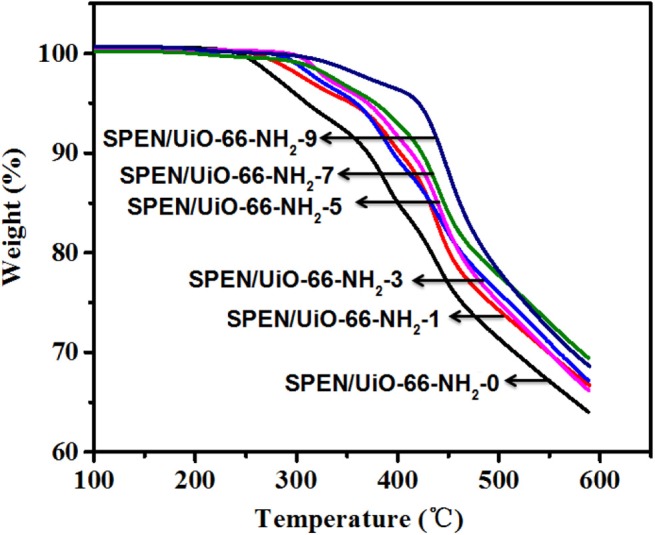
The thermal degradation (TGA) curve of SPEN/UiO-66-NH_2_-x covalent-ionically cross-linked composite membrane.

The thermal stability of the prepared SPEN/UiO-66-NH_2_-x covalent-ionically cross-linked composite membrane was studied by TGA in nitrogen atmosphere, as shown in Figure 4. The TGA curves of all SPEN/UiO-66-NH_2_-x composite membranes show two decomposition patterns. The first decomposition range can be attributed to the thermal decomposition of sulfonic groups, and the second decomposition range can be attributed to the degradation of the main chain of SPEN. Furthermore, the initial decomposition temperature of SPEN/UiO-66-NH_2_-0 composite membrane is about 260°C, which is much higher than that of Nafion 117. With the addition of UiO-66-NH_2_, the thermal decomposition temperature of the SPEN/UiO-66-NH_2_-x composite membranes increases greatly, and the initial decomposition temperature of all the composite membranes is higher than 290°C. It can be attributed that: (1) After the formation of covalent-ionically cross-linking between UiO-66-NH_2_ and SPEN, the movement of molecular chains and sulfonic acid groups was limited, thus the thermal stability of the SPEN/UiO-66-NH_2_-x composite membranes could be improved accordingly; (2) as a kind of functional nanoparticles, the interaction between UiO-66-NH_2_ and molecular chains hinders the mobilities of the chains, and delays the degradation of SPEN molecular chains, thus improving the thermal stability of the SPEN/UiO-66-NH_2_-x composite membranes.

### Mechanical Properties

**Figure 5:**
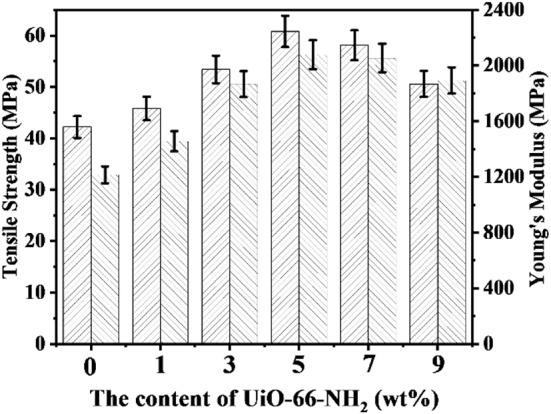
The mechanical properties of SPEN/UiO-66-NH_2_-x covalent-ionically cross-linked composite membrane.

The mechanical properties of proton exchange membranes (PEMs) directly determines whether the PEMs can be used for long-term operation in fuel cells. The mechanical properties of SPEN/UiO-66-NH_2_-x composite membranes in wet state are listed in Figure 5. The tensile strength and Young's modulus of SPEN/UiO-66-NH_2_-x composite membranes are 42.2–60.8 MPa and 1,215–2,078 MPa, respectively. With the addition of UiO-66-NH_2_, all composite membranes demonstrated enhanced mechanical properties. This is mainly due to the covalent-ionically cross-linking between UiO-66-NH_2_ and SPEN, which results in the lower mobility of SPEN molecular chains in a certain range. Both the tensile strength and Young's modulus of SPEN/UiO-66-NH_2_-x composite membranes show an increasing trend with the addition of UiO-66-NH_2_ <5 wt%. However, while the content exceeds 5 wt%, the mechanical properties begin to decrease, which is determined by the dispersion of UiO-66-NH_2_. As mentioned above by SEM, UiO-66-NH_2_ has good compatibility and good dispersion with the SPEN matrix as the content of UiO-66-NH_2_ below 5 wt%, which can enhance the mechanical properties of the SPEN/UiO-66-NH_2_-x composite membranes. While the addition exceeds 5 wt%, UiO-66-NH_2_ is prone to agglomeration, which resulting in defects in the SPEN/UiO-66-NH_2_-x composite membranes.

### Ion Exchange Capacity, Water Uptake, and Swelling Ratio

**Figure 6:**
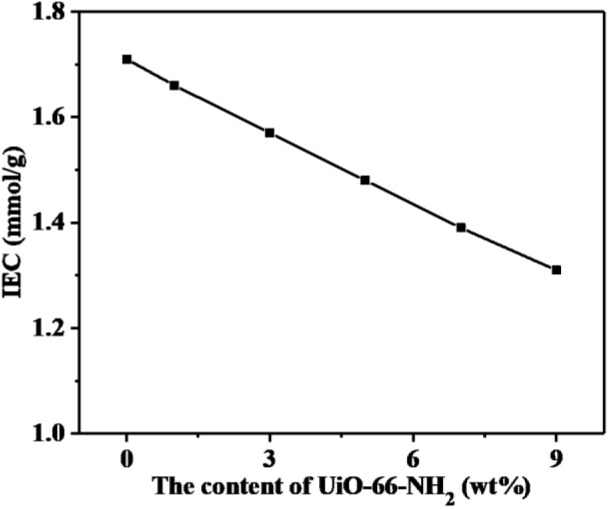
The ion exchange capacity (IEC) values of SPEN/UiO-66-NH_2_-x covalent-ionically cross-linked composite membrane.

The ion exchange capacity (IEC) values represent the amount of hydrogen ions that can be replaced in proton exchange membranes, which plays an important role to the water uptake, proton conductive, and dimensional stability of membranes. As shown in Figure 6, with the increase of UiO-66-NH_2_ addition, the IEC value of SPEN/UiO-66-NH_2_-x composite membranes decreased from 1.71 to 1.31 mmol/g. The main reasons are as follows: (1) The addition of UiO-66-NH_2_ to the SPEN matrix diluted the concentration of sulfonic groups in the composite membranes to a certain extent. (2) The addition of UiO-66-NH_2_ can cause covalent-ionically cross-linking reaction with carboxyl and sulfonic groups under certain heat treatment, which consumes the carboxyl and sulfonic groups, and the cross-linking network limits the replacement of H^+^ by Na^+^, thus leading to the decrease of IEC value of the composite membrane.

**Figure 7:**
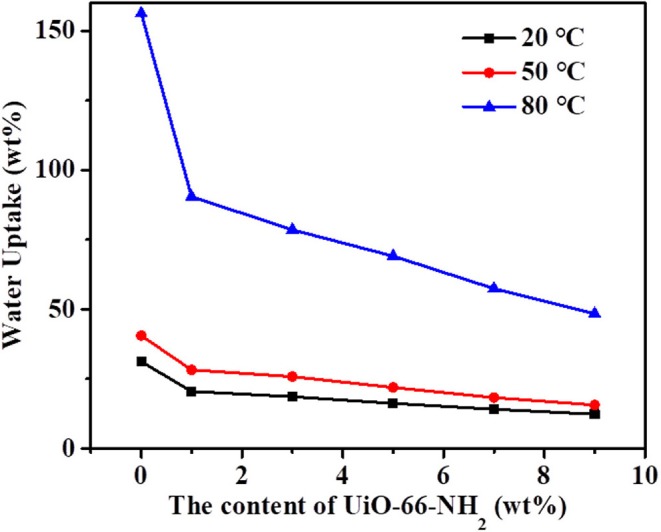
The water uptake of SPEN/UiO-66-NH_2_-x covalent-ionically cross-linked composite membrane.

The water uptake of the PEMs has an important influence on the proton transport mechanism and methanol permeability. It is also closely related to the dimensional stability and mechanical properties of the proton exchange membrane. Therefore, the proton exchange membrane must have a proper water uptake. The water uptake of SPEN/UiO-66-NH_2_-x composite membrane at corresponding temperatures is shown in Figure 7. The water uptake of SPEN/UiO-66-NH_2_-x composite membrane increases with the increasing temperature. For example, at room temperature, the water uptake of SPEN/UiO-66-NH_2_-0 composite membranes is 31.2 wt%, and at 80°C, the water uptake of SPEN/UiO-66-NH_2_-0 composite membranes is 156.2 wt%. It was also observed that the water uptake of the SPEN/UiO-66-NH_2_-x composite membranes decreased gradually with the increasing of UiO-66-NH_2_ content. For example, at room temperature, the water uptake of SPEN/UiO-66-NH_2_-x composite membranes decreased from 31.2 to 12.3 wt% with the increase of UiO-66-NH_2_ content from 0 to 9 wt%. The reasons for this phenomenon can be attributed to two aspects: (1) After the addition of UiO-66-NH_2_, the covalent-ionically cross-linking between UiO-66-NH_2_ and SPEN was formed. In addition, UiO-66-NH_2_ as a filler could also restrict the molecular chain movement of SPEN. These two effects synergistically reduced the free volume of the composite membrane, thus resulting in the water uptake of the composite membrane decreased correspondingly. (2) The UiO-66-NH_2_ filler has lower IEC value than SPEN leads to the decreasing water uptake with the increasing of UiO-66-NH_2_ content after covalent-ionically cross-linking with the matrix.

**Figure 8:**
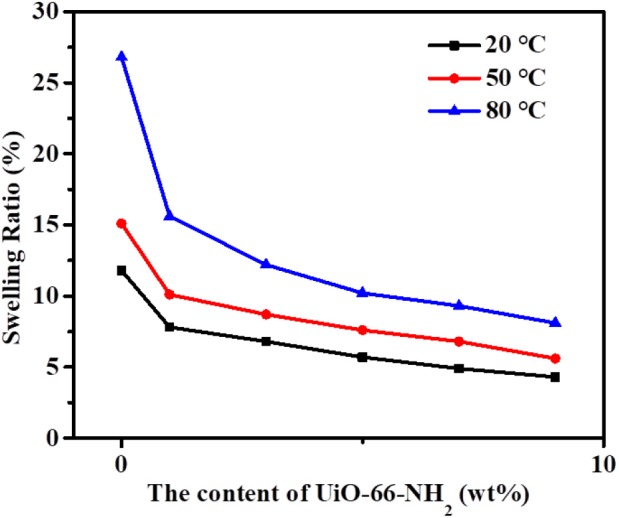
The swelling ratio of SPEN/UiO-66-NH_2_-x covalent-ionically cross-linked composite membrane.

From Figure 8, it can be seen that the swelling ratio of SPEN/UiO-66-NH_2_-x composite membranes at different temperatures shows a similar trend to that of the above-mentioned water uptake; that is, the swelling ratio increases with the increasing temperature and decreases with the increasing filler content. For example, at room temperature, the swelling rate of SPEN/UiO-66-NH_2_-0 composite membrane is 11.8%, while that of SPEN/UiO-66-NH_2_-9 composite membrane is 4.3%. When the temperature is raised from 20 to 80°C, the swelling ratio of SPEN/UiO-66-NH_2_-0 composite membrane is 26.8%, while that of SPEN/UiO-66-NH_2_-9 composite membrane is 8.1%. The above results show that the formation of covalent-ionically cross-linking can significantly improve the dimensional stability of SPEN.

### Proton Conductivity

**Figure 9:**
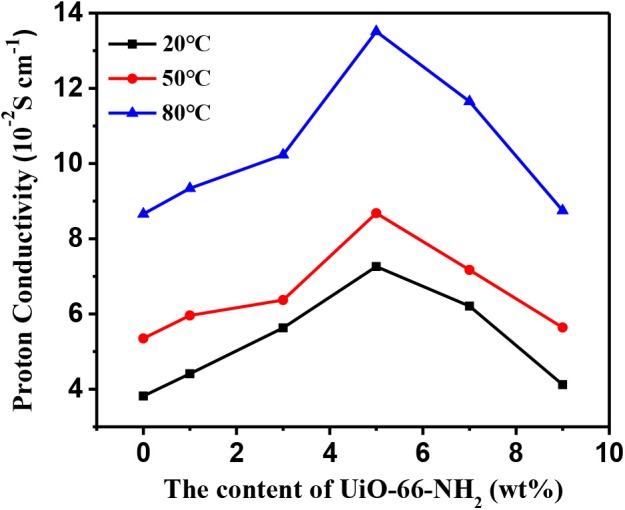
The proton conductivity of SPEN/UiO-66-NH_2_-x covalent-ionically cross-linked composite membrane.

**Figure 10:**
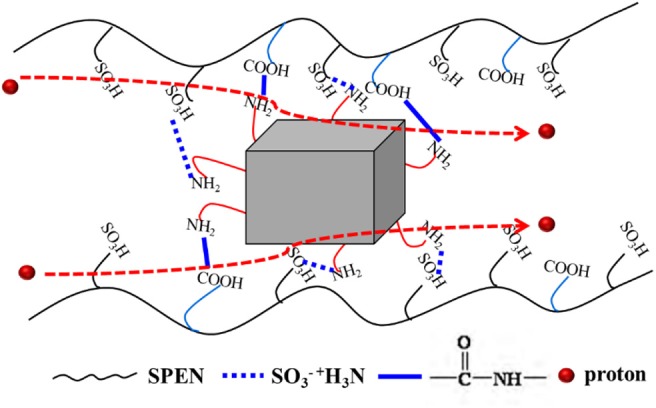
The proposed proton transport mechanism in SPEN/UiO-66-NH_2_-x covalent-ionically cross-linked composite membrane.

The effects of temperature and UiO-66-NH_2_ contents on the proton conductivity of SPEN/UiO-66-NH_2_-x covalentionically cross-linked composite membrane are shown in Figure 9 and Table 1. Firstly, the proton conductivity of the SPEN/UiO-66-NH_2_-x composite membranes increases with the increasing temperature. For example, the proton conductivity of SPEN/UiO-66-NH_2_-5 composite membrane is 7.26 × 10^−2^ S·cm^−1^ at 20°C, while the proton conductivity of SPEN/UiO-66-NH_2_-5 composite membrane is 13.51 × 10^−2^ S·cm^−1^ at 80°C. This can be attributed to the fact that temperature increases can promote the mobility of water molecules and polymer chains, thus improving the migration rate of hydrated protons. At all temperatures, the proton conductivity of the SPEN/UiO-66-NH_2_-x composite membranes increased first and then decreased with the increasing UiO-66-NH_2_ content. These phenomena can be explained by the illustration of Figure 10. When the UiO-66-NH_2_ content is <5 wt%, the increase of proton conductivity can be attributed to the fact that the amino groups in the UiO-66-NH_2_ framework are good proton acceptors/donors. When the content of UiO-66-NH_2_ is ≤ 5 wt%, it can be uniformly dispersed and cause the change of sulfonic groups at the interface between UiO-66-NH_2_ and SPEN matrix. The sulfonic groups interact with the basic amino group to form acid–base proton pairs, with the aid of water molecule, protons are continuously transferred between amino group and sulfonic groups in the membrane by acid–base interaction and Grotthuss mechanism. In this process, the formation and breakage of hydrogen bond network structure are alternately carried out. In conclusion, UiO-66-NH_2_ can enhance the proton conduction of the composite membrane by increasing the density of proton conduction groups at the interface of UiO-66-NH_2_/SPEN under the condition of good rational dispersion; the other part of protons can combine with free water molecules to form hydrated protons (H_3_O^+^), which can transport through a continuous ion transport channel. Therefore, the addition of UiO-66-NH_2_ into SPEN could significantly enhance proton transport. However, when the content of UiO-66-NH_2_ continued to increase in the composite membrane, the proton conductivity of the composite membrane began to show a downward trend, especially that of SPEN/UiO-66-NH_2_-9 composite membrane, which decreased to 4.12 × 10^−2^ S·cm^−1^ (at room temperature). This is mainly attributed to the aggregation of UiO-66-NH_2_ when the content is too high, which makes its dispersion in composite membranes become worse. In addition, the high content of UiO-66-NH_2_ leads to the decrease of IEC value of composite membranes. In summary, it is more appropriate to control the content of UiO-66-NH_2_ at about 5 wt%.

**Table 1:** The proton conductivity, methanol permeability, and selectivity of SPEN/UiO-66-NH_2_-x covalent-ionically cross-linked composite membrane.

**Membranes**	**Proton conductivity** **(10^−2^ S·cm^−1^)**			**Methanol permeability** **(10^−7^ cm^2^·s^−1^)**	**Selectivity** **(10^5^ S·s·cm^−3^)**
	**20^**°**^C**	**50^**°**^C**	**80^**°**^C**		
SPEN/UiO-66-NH_2_-0	3.82	5.35	8.66	3.12	1.22
SPEN/UiO-66-NH_2_-1	4.41	5.96	9.34	2.65	1.66
SPEN/UiO-66-NH_2_-3	5.63	6.37	10.23	1.85	3.04
SPEN/UiO-66-NH_2_-5	7.26	8.68	13.51	1.13	6.42
SPEN/UiO-66-NH_2_-7	6.21	7.17	11.65	1.04	5.97
SPEN/UiO-66-NH_2_-9	4.12	5.64	8.75	0.85	4.84
Nafion117	6.4	–	–	14.1	0.45

### Methanol Permeability and Selectivity

**Figure 11:**
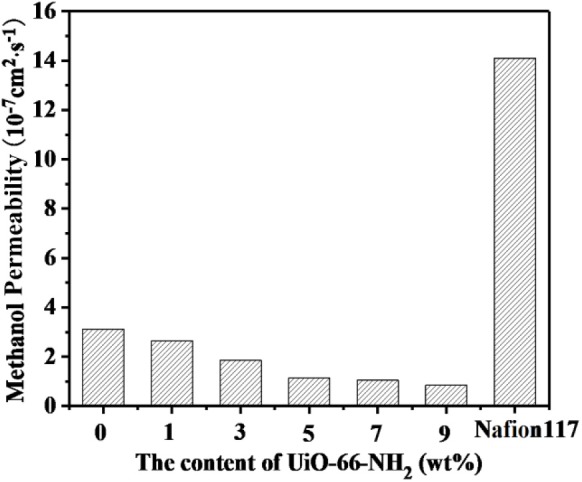
The methanol permeability of SPEN/UiO-66-NH_2_-x covalent-ionically cross-linked composite membrane.

Methanol permeability from anode to cathode can lead to fuel loss, polarization of fuel cell electrodes and significant reduction of fuel cell efficiency, and even catalyst poisoning. Therefore, in DMFCs, proton exchange membranes must possess both excellent proton conductivity and methanol resistance. The methanol permeability of SPEN/UiO-66-NH_2_-x composite membranes is listed in Table 1 and Figure 11. The methanol permeability of the SPEN/UiO-66-NH_2_-x composite membranes decreased gradually with the increasing of UiO-66-NH_2_ content. The methanol permeability of the SPEN/UiO-66-NH_2_-x composite membranes ranged from 3.2 × 10^−7^S·cm^2^·s^−1^ to 0.85 × 10^−7^ S·cm^2^·s^−1^, and the SPEN/UiO-66-NH_2_-9 composite membranes had the lowest methanol permeability. This is mainly due to: (1) UiO-66-NH_2_ covalent-ionically cross-linking with carboxyl and sulfonic groups will form a three-dimensional cross-linking network in the membranes. The cross-linking network restricts the movement of SPEN molecular chains, thus making the membranes more compact and the methanol permeability of the SPEN/UiO-66-NH_2_-x composite membranes significantly reduced. (2) The interaction between the nitrile group of SPEN and UiO-66-NH_2_ further inhibits the mobility of the polymer chain and reduces the free volume of the composite membrane. (3) The transport of methanol in the membrane is similar to that of proton transport mechanism, which requires hydrophilic ion channels in the membrane. The barrier effect of UiO-66-NH_2_ in the proton transport channel can increase the curvature of the transport channel, thus prolonging the methanol diffusion pathway and reducing the methanol permeability. (4) UiO-66-NH_2_ can trap methanol in its pores, thus inhibiting the methanol permeability.

**Figure 12:**
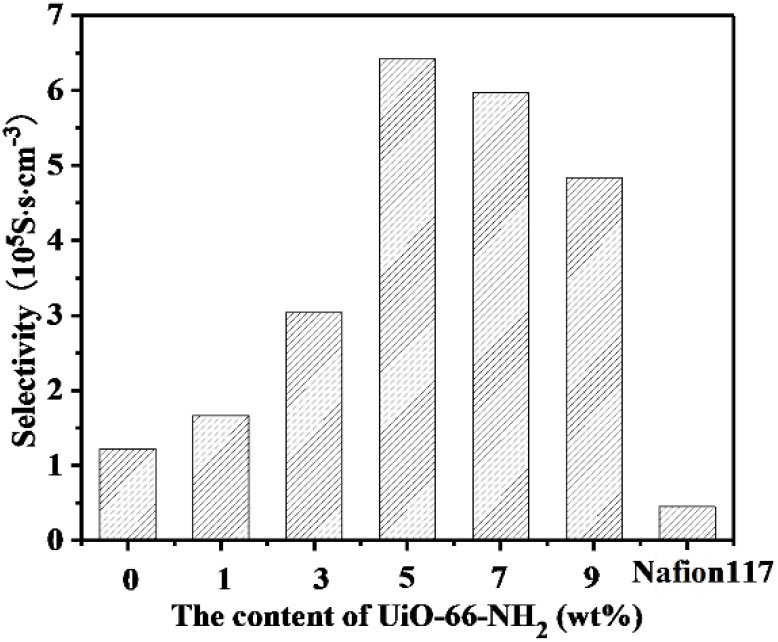
The selectivity of SPEN/UiO-66-NH_2_-x covalent-ionically cross-linked composite membrane.

As shown in Figure 12 and Table 1, with the increasing addition of UiO-66-NH_2_, the selectivity (which is calculated by the ratio of proton conductivity to methanol permeability) of all SPEN/UiO-66-NH_2_-x composite membranes are improved compared with pure SPEN membranes. This is mainly due to the increase of proton conductivity and methanol resistance of SPEN/UiO-66-NH_2_-x composite membranes. However, the selectivity of the SPEN/UiO-66-NH_2_-x composite membranes increased first and then decreased with the increasing of UiO-66-NH_2_ content. This was mainly due to the dispersion, interface connectivity, and compatibility of UiO-66-NH_2_ in the SPEN matrix. It is for the fact that when the UiO-66-NH_2_ content is below 5 wt%, the methanol permeability and the proton conductivity of SPEN/UiO-66-NH_2_-x increase simultaneously. Thus, the selectivity of SPEN/UiO-66-NH_2_-x presents a growing trend with increasing x. However, when the content of UiO-66-NH_2_ exceeds 5 wt%, the increased cross-linking structure makes the membrane highly dense, which results in more significant decrease of proton conductivity than that of methanol permeability. Thus, the selectivity of SPEN/UiO-66-NH_2_-x membranes presents a downward trend. The UiO-66-NH_2_ addition can increase the proton conductivity and methanol resistance of SPEN/UiO-66-NH_2_-x; however, excessive UiO-66-NH_2_ may lead to the agglomeration which decreases the proton conductivity in the SPEN/UiO-66-NH_2_-x membranes and leads to the reduction of overall performance. The SPEN/UiO-66-NH_2_-5 composite membrane has the highest selectivity, which is 6.42 × 10^5^ S·s·cm^−3^, 14.3-times higher than that of Nafion 117.

## Conclusion

In this manuscript, we have successfully achieved a high-performance SPEN/UiO-66-NH_2_-x covalent-ionically cross-linked composite membrane by doping UiO-66-NH_2_ (a kind of newly emerging porous materials with solution processability) into SPEN. The structure and morphology of large-scale synthesized UiO-66-NH_2_ were characterized by XRD, SEM, TEM, and BET. Simultaneously, the obtained SPEN/UiO-66-NH_2_-x covalent-ionically cross-linked composite membranes displays excellent thermal stability and dimensional stability. The as-prepared SPEN/UiO-66-NH_2_-x composite membranes display excellent proton conductivity compared with that of recast SPEN. The enhanced proton conductivity can be attributed to the construction of ionic clusters and well-connected ionic nanochannels along the interface between UiO-66-NH_2_-x and SPEN matrix via molecular interactions. Meanwhile, the methanol permeability of SPEN/UiO-66-NH_2_-x covalent-ionically cross-linked composite membrane is well-suppressed due to the barrier effect of cross-linking and UiO-66-NH_2_-x. At the same time, the selectivity of SPEN/UiO-66-NH_2_-x also increased significantly as the UiO-66-NH_2_-x was added. The SPEN/UiO-66-NH_2_-5 composite membrane exhibits the highest selectivity of 6.42 × 10^5^ S·s·cm^−3^, 14.3-times higher than that of Nafion 117. All the above results indicate that preparation of SPEN/UiO-66-NH_2_-x covalent-ionically cross-linked composite membranes is an efficient method to modify PEMs with improved proton-transfer and methanol resistant properties.

## Data Availability Statement

The datasets generated for this study are available on request to the corresponding author.

## Author Contributions

PZ: writing—original draft preparation, conceptualization, methodology, investigation, and data curation. YZ: writing—review and editing. ZL, DW, and YM: data curation. QL: validation.

### Conflict of Interest

The authors declare that the research was conducted in the absence of any commercial or financial relationships that could be construed as a potential conflict of interest.
